# Relating the molecular phenotype of ulcerative colitis to the clinical course

**DOI:** 10.1038/s41598-025-90618-8

**Published:** 2025-03-11

**Authors:** Katelynn S. Madill-Thomsen, Jeffery M. Venner, Denise E. Parsons, Konrad S. Famulski, Aducio L. Thiesen, Sami Hoque, Karen I. Kroeker, Karen Wong, Farhad Peerani, Levinus A. Dieleman, Frank Hoentjen, Daniel C. Baumgart, Philip F. Halloran, Brendan P. Halloran

**Affiliations:** 1 Department of Medicine, University of Alberta, Edmonton, AB Canada; 2 Alberta Transplant Applied Genomics Center, University of Alberta, Edmonton, AB Canada; 3 Cedars-Sinai Medical Center, Los Angeles, CA USA; 4 Department of Medicine, Division of Gastroenterology, University of Alberta, 8540 – 112 Street NW, Edmonton, AB T6G 2X8 Canada; 5 Department of Laboratory Medicine and Pathology, University of Alberta, Edmonton, AB Canada

**Keywords:** Inflammatory bowel disease, Molecular classifier, Machine learning, Molecular landscape, Disease activity, Gastroenterology, Medical research, Molecular medicine, Pathogenesis, Gene ontology, Microarrays, Predictive medicine, Adaptive immunity, Autoimmunity, Inflammation, Translational immunology, Transcriptomics, Biomarkers, Diagnostic markers, Prognostic markers

## Abstract

The expanding portfolio of targeted therapies for ulcerative colitis (UC) suggests that a more precise approach to defining disease activity will aid clinical decision-making. This prospective study used genome-wide microarrays to characterize gene expression in biopsies from the most inflamed colon segments from patients with UC and analyzed associations between molecular changes and short-term outcomes while on standard-of-care treatment. We analyzed 141 biopsies—128 biopsies from 112 UC patients and 13 biopsies from eight inflammatory bowel disease unclassified (IBDU) patients. Endoscopic disease was associated with expression of innate immunity transcripts, e.g. complement factor B (CFB); inflammasome genes (ZBP1 and PIM2); calprotectin (S100A8 and S100A9); and inflammation-, injury-, and innate immunity-associated pathway analysis terms. A cross-validated molecular machine learning classifier trained on the endoscopic Mayo subscore predicted the endoscopic Mayo subscore with area-under-the-curve of 0.85. A molecular calprotectin transcript score showed strong associations with fecal calprotectin and the endoscopic Mayo subscore. Logistic regression models showed that molecular features (e.g. molecular classifier and molecular calprotectin scores) improved the prediction of disease progression over conventional, clinical features alone (e.g. total Mayo score, fecal calprotectin, physician global assessment). The molecular features of UC showed strong correlations with disease activity and permitted development of machine-learning predictive disease classifiers that can be applied to expanded testing in diverse cohorts.

**Supplementary Information:**

The online version contains supplementary material available at 10.1038/s41598-025-90618-8.

## Background & aims

The goal of treatment in ulcerative colitis (UC) is to suppress disease activity and improve patient outcomes. This is balanced against the risk of medication side effects and over-treatment that can lead to adverse outcomes and unnecessary expense. Currently, disease assessment is hindered by limitations of current clinical classification systems^1^, and the suboptimal understanding of the relationship between disease phenotype and patient outcomes^2^. Conventional assessment of UC uses a combination of clinical parameters (e.g. partial Mayo score), biochemical markers (e.g. fecal calprotectin), and endoscopic evaluation (e.g. endoscopic Mayo subscore)^2^. Fecal calprotectin is non-invasive but has limited sensitivity (78%) and specificity (73%) for prediction of patient outcomes^3^. The endoscopic Mayo subscore is widely accepted^4,5^ but has substantial intra- and interobserver variability^6^ and the categorical scores (0–3) have limited ability to reflect disease heterogeneity. Histology scoring (i.e. Geboes Score) is labor-intensive, requires specialized pathologists, and is also subject to substantial interobserver variability^7,8^.

There is an unmet need that can be addressed using molecular tools capable of assessing UC and extending the current classifications via mucosal biopsies obtained during endoscopy. Many studies have focused on assessing differentially-expressed genes between UC biopsies and normal colon tissue^9–11^, or severe UC versus mild/moderate UC using spatially-resolved single-cell sequencing^12^, but faced difficulties in predicting disease severity and/or were under powered. Recent studies have also speculated at the interplay between ‘epithelially-activated’ and ‘immune-activated’ disease to explain the varying response to biologics such as golimumab, infliximab, and vedolizumab^13^. We previously used microarrays to make genome-wide measurements of gene expression in UC biopsies^14^, showing a large-scale disturbance involving inflammation and parenchymal injury and dedifferentiation, with similarities to transcript changes seen in other chronic inflammatory conditions^15–19^. Expression of transcript sets previously associated with T cell activity and parenchymal injury correlated with the endoscopic Mayo subscore and the presence of lamina propria lymphoplasmacytic infiltrate in colon biopsies^14^.

**Fig. 1:**
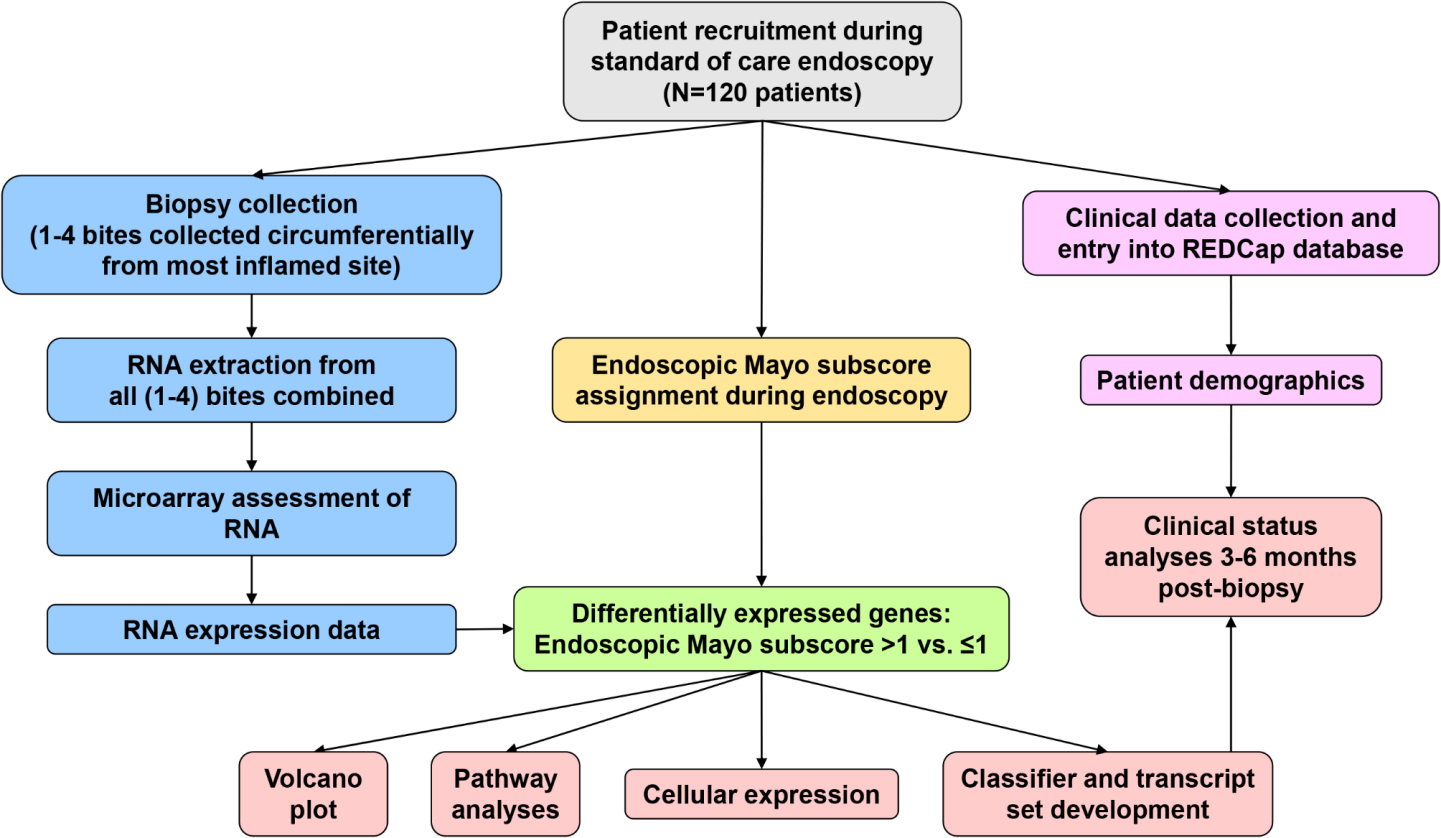
Study flowchart.

The present study aimed to develop a molecular scoring system that would add insight and value to existing systems. We selected microarrays as the molecular platform rather than sequencing because microarrays are highly established and can be standardized for operation in testing centers and interpreted by machine learning algorithms^20,21^. We documented the molecular changes that correlate with UC disease activity (i.e. endoscopic Mayo subscores > 1 vs. ≤1) to understand the biological mechanisms operating at the mucosal level, developed cross-validated molecular classifiers to reduce UC disease activity to a molecular scoring system that would provide continuous numbers rather than categorical classes, and evaluated the relationship of these scores to future disease activity and clinical outcomes. An overview of the study workflow is shown in Fig. 1.

## Results

### Patient population and demographics

141 colon biopsies from 120 patients with UC based on current guidelines^22,23^ were prospectively collected from two quaternary academic referral centers in Canada and the United States. A small number of biopsies (*N* = 13 biopsies from 8 patients) initially labeled as UC had some indeterminate features (such as non-confluent disease on endoscopy and minimal bleeding) on review. Our review relabeled these biopsies as inflammatory bowel disease unclassified (IBDU). These patients were originally characterized as UC previously and were clinically managed as such. Table 1 summarizes patient and biopsy demographics. Of note, biopsies used in this study were collected from the most inflamed area, as described in the methods.

**Table 1:** Demographics of the patients (*N* = 120) and biopsies (*N* = 141).

Patient characteristics (*N* = 120)	UC patients (*N* = 112)	IBDU patients (*N* = 8)	
Mean patient age (median, range) in years	43 (41, 19–83)	40 (39, 31–50)	
Patient gender (% male)	51 (54% male, 17 NA)	4 (50% male, 2 NA)	
Diagnosis			
Ulcerative colitis (UC)	112	0	
Inflammatory bowel disease unclassified (IBDU)	0	8	
Centers			
University of Alberta Hospital – Edmonton	95	6	
Cedars-Sinai Medical Center – Los Angeles	17	2	

Biopsy characteristics (*N* = 141)	UC biopsies (*N* = 128)	IBDU biopsies (*N* = 13)	
Median time from index to last known follow-up in months (mean)	5.1 (4.7)	6.2 (6.2)	
Endoscopy and biopsy indication			
New diagnosis	5	-	
Disease activity assessment	73	6	
Dysplasia screening	6	1	
Fecal microbiota transplant	1	-	
Cancer screening	2	-	
NA	41	6	
Disease extent			
Remission	57	5	
Proctitis	11	1	
Left-sided	26	4	
Extensive	4	-	
Pancolitis	22	1	
NA	8	2	
Endoscopic Mayo subscore at biopsy location			
0	24	1	
1	30	3	
2	45	4	
3	29	5	
Treatment regimen at the time of the biopsy^a^			
5-aminosalicylic acid (topical or oral)	62	4	
Biologic	27	3	
Immunomodulator^b^	30	4	
Infliximab	16	1	
Adalimumab	4	2	
Golimumab	2	-	
Vedolizumab	4	-	
Other therapy	8	2	

NA, not available; UC, ulcerative colitis; IBDU, IBD unclassified.

^a^Patients on a combination of therapies were not excluded. Biopsies from Cedars-Sinai had unavailable treatment information, accounting for 19 patients.

^b^Immunomodulator includes thiopurines (azathioprine, 6-mercaptopurine) and methotrexate.

### Transcripts associated with UC disease activity

**Fig. 2:**
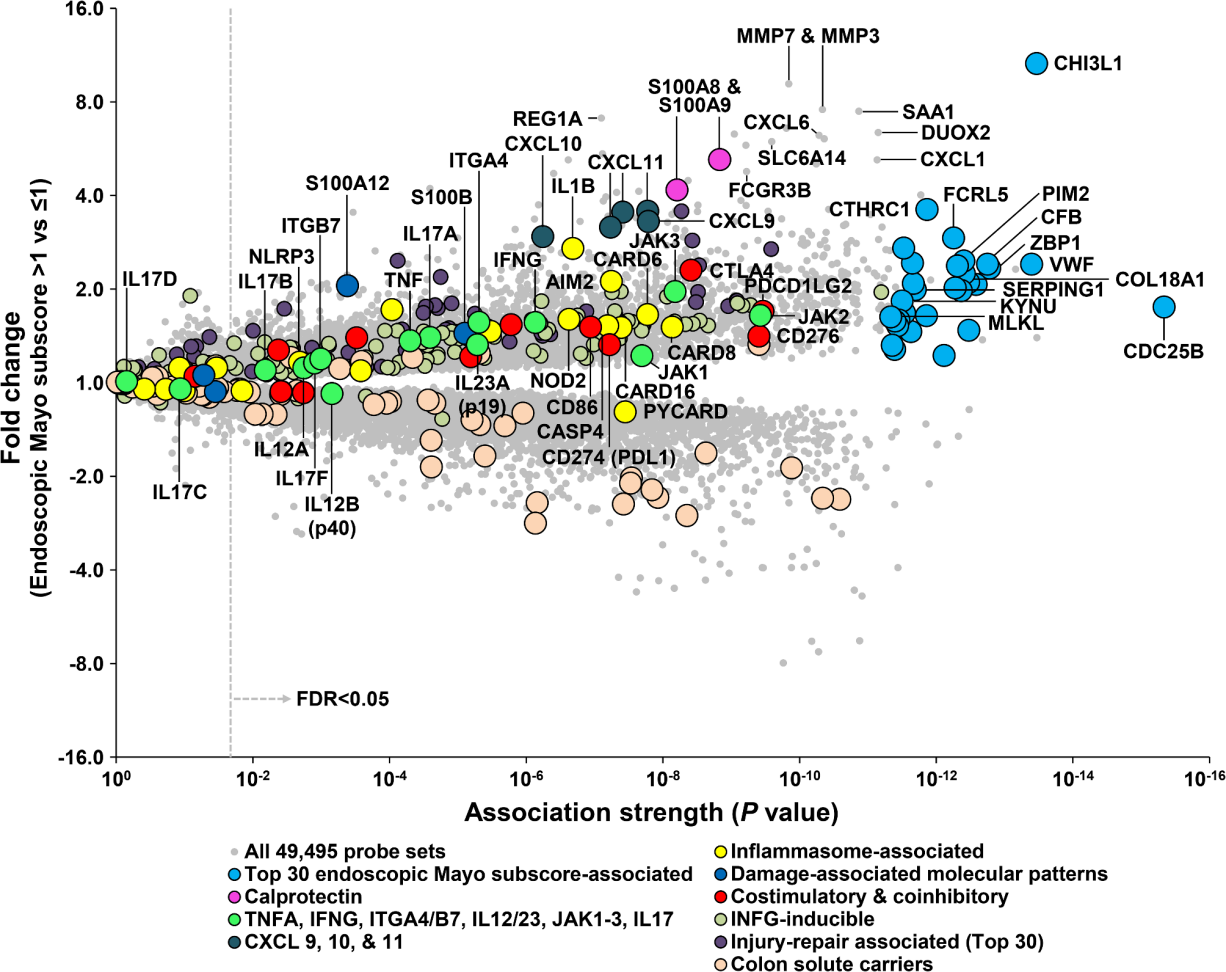
Molecular landscape of ulcerative colitis in 128 biopsies as shown by a volcano plot. Probe sets towards the upper right have high association and fold change, indicating a strong relationship with UC disease activity as represented by increased endoscopic Mayo subscore (> 1 vs. ≤ 1). Probe sets towards the middle and further left have moderate to lower associations and fold change, indicating a lack of relationship with UC activity. The FDR (false discovery rate) of < 0.05 is indicated by the grey, dashed line. Transcripts of interest were annotated. Abbreviations: UC, ulcerative colitis.

Total RNA was extracted from biopsies and processed for Affymetrix PrimeView GeneChip microarrays (see methods). We analyzed the transcripts associated with UC disease activity, high (> 1) vs. low (≤ 1) endoscopic Mayo subscore, by ranking each transcript’s association strength (unadjusted *P* value) within the UC biopsies (*N* = 128). Molecules of special interest in UC were flagged (Fig. 2). At *P* < 0.05, we found 21,768 probe sets (11,658 unique transcripts) with increased or decreased expression in association with a high endoscopic Mayo subscore.

The top 30 transcripts (by *P* value) increased in UC with a high endoscopic Mayo subscore are shown as blue dots in Fig. 2. The top transcript was cell division cycle 25B (CDC25B, unadjusted *P* = 4.6 × 10^−16^), a phosphatase and regulator of G2/M phases of the cell cycle with broad expression^24^. Other top transcripts included components of innate immunity expressed in macrophages e.g. complement factor B (CFB, *P* = 1.8 × 10^−13^) and macrophage gene CHI3L3; regulators of the NLRP3 inflammasome, e.g. serine/threonine kinase PIM2 (*P* = 3.9 × 10^−13^); and markers of endothelial injury and matrix remodeling, e.g. lysyl oxidase-like 2 (LOXL2, *P* = 4.3 × 10^−13^). A number of these transcripts including VWF^25^, CFB^26^, and LOXL2^27^ have previously been shown to be related to UC activity. Calprotectin transcripts S100A8 and S100A9 were also associated with disease activity (*P* = 1.5 × 10^−9^ and 6.2 × 10^−9^), but not within the top 30.

Targets of advanced therapy used in UC were associated with disease activity (Fig. 2, green dots): tumor necrosis factor alpha (TNF, *P* = 5.0 × 10^−5^); IL12B (p40 subunit, *P* = 6.9 × 10^−4^), which forms heterodimers with IL12A (*P* = 1.8 × 10^−3^) or IL23A (p19 subunit, *P* = 5.2 × 10^−6^); integrin alpha 4 (ITGA4, *P* = 4.9 × 10^−6^) and ITGB7 (*P* = 1.0 × 10^−3^); and Janus kinase JAK2 (*P* = 3.7 × 10^−10^), JAK3 (*P* = 6.6 × 10^−9^), and JAK1 (*P* = 2.0 × 10^−8^).

Inflammasome transcripts associated with UC disease activity included caspase recruitment domain family, member 8 (CARD8, *P* = 7.3 × 10^−9^), nucleotide-binding oligomerization domain containing 2 (NOD2, *P* = 2.4 × 10^−7^), NLRP3 (*P* = 2.1 × 10^−3^) and NLRP9 (*P* = 3.0 × 10^−2^) transcripts (Fig. 2, yellow dots), significant in UC for their relationship to pathogenesis but also their potential role in colonocyte wound repair^28,29^. Three inflammasome transcripts implicated in epithelial stem cell (EpSC) reprogramming during injury^30,31^ were moderately associated with the endoscopic Mayo subscore: interferon-inducible protein (AIM2, *P* = 5.7 × 10^−8^), interleukin 1 beta (IL1B, *P* = 2.1 × 10^−7^), and caspase 1 (CASP1, *P* = 3.3 × 10^−6^).

Solute carrier transcripts highly expressed in normal colon^14^ were decreased in active UC e.g. SLA26A2 (involved in matrix organization) and bicarbonate transporter SLC4A4 (Fig. 2, tan dots).

Details of the top 30 transcripts increased in biopsies with high endoscopic Mayo subscores are shown in Table 2, and the top 30 transcripts with decreased expression in biopsies with high endoscopic Mayo subscore are outlined in Table 3.

**Table 2:** Top 30 unique transcripts by *P* value with increased expression in biopsies with endoscopic Mayo subcore >1 vs. ≤1 (*N *= 128 biopsies).

*P* value	Adjusted *P* value	Gene Symbol	Gene Name	Expression in biopsies			Cellular Expression in Colon tissue per Human Protein Atlas^a^
				Mayo > 1	Mayo ≤ 1	Control	
4.61 × 10^−16^	2.28 × 10^−11^	CDC25B*	Cell division cycle 25B	617	353	292	colonocytes, T cells, B cells
3.40 × 10^−14^	4.99 × 10^−10^	CHI3L1*	Chitinase 3-like 1	1125	106	47	NA
4.03 × 10^−14^	4.99 × 10^−10^	VWF*	von Willebrand factor	549	228	180	Granulocytes
1.65 × 10^−13^	1.25 × 10^−9^	ZBP1*	Z-DNA binding protein 1	172	74	63	Colonocytes
1.77 × 10^−13^	1.25 × 10^−9^	CFB*	Complement factor B	1861	774	462	NA
2.61 × 10^−13^	1.38 × 10^−9^	COL18A1*	Collagen, type XVIII, alpha 1	287	139	126	Granulocytes
3.33 × 10^−13^	1.38 × 10^−9^	TRAM2*	Translocation associated membrane protein 2	981	665	630	Intestinal endocrine cells, undifferentiated cells, mucus-secreting cells, B cells, colonocytes
3.36 × 10^−13^	1.38 × 10^−9^	FER1L4*	Fer-1-like 4 (C. elegans) pseudogene	138	66	76	NA
3.90 × 10^−13^	1.39 × 10^−9^	LOC100130872*	Uncharacterized LOC100130872	1252	558	529	NA
3.93 × 10^−13^	1.39 × 10^−9^	PIM2*	Pim-2 oncogene	913	371	330	B cells, undifferentiated cells, T cells, intestinal endocrine cells
4.28 × 10^−13^	1.41 × 10^−9^	LOXL2*	Lysyl oxidase-like 2	395	200	139	B cells, undifferentiated cells
4.90 × 10^−13^	1.43 × 10^−9^	OLFML2B*	Olfactomedin-like 2B	177	74	71	B cells
5.28 × 10^−13^	1.45 × 10^−9^	TGM2*	Transglutaminase 2 (C polypeptide, protein-glutamine-gamma-glutamyltransferase)	127	63	64	Colonocytes
5.57 × 10^−13^	1.45 × 10^−9^	FCRL5*	Fc receptor-like 5	300	103	110	B cells, undifferentiated cells
7.66 × 10^−13^	1.90 × 10^−9^	NRD1*	Nardilysin (N-arginine dibasic convertase)	621	508	489	NA
1.36 × 10^−12^	2.61 × 10^−9^	CTHRC1*	Collagen triple helix repeat containing 1	599	166	149	Undifferentiated cells
1.38 × 10^−12^	2.61 × 10^−9^	RPS6KA2*	Ribosomal protein S6 kinase, 90 kDa, polypeptide 2	259	157	143	Colonocytes, intestinal endocrine cells, undifferentiated cells, mucus-secreting cells
2.02 × 10^−12^	3.44 × 10^−9^	SERPING1*	Serpin peptidase inhibitor, clade G (C1 inhibitor), member 1	418	210	202	NA
2.15 × 10^−12^	3.52 × 10^−9^	ELL2*	Elongation factor, RNA polymerase II, 2	576	277	262	Granulocytes, intestinal endocrine cells, B cells, colonocytes
2.23 × 10^−12^	3.52 × 10^−9^	COL4A1*	Collagen, type IV, alpha 1	964	398	302	Undifferentiated cells, mucus-secreting cells
2.35 × 10^−12^	3.52 × 10^−9^	ODF3B	Outer dense fiber of sperm tails 3B	235	161	141	B cells, colonocytes, intestinal endocrine cells, undifferentiated cells, mucus-secreting cells
2.90 × 10^−12^	4.10 × 10^−9^	IFITM2	Interferon induced transmembrane protein 2	1480	884	769	T cells, granulocytes, B cells, undifferentiated cells
2.99 × 10^−12^	4.11 × 10^−9^	MZB1	Marginal zone B and B1 cell-specific protein	1987	733	779	Undifferentiated cells
3.15 × 10^−12^	4.21 × 10^−9^	KYNU	Kynureninase	169	93	85	B cells, undifferentiated cells, colonocytes
3.51 × 10^−12^	4.26 × 10^−9^	GJA4	Gap junction protein, alpha 4, 37 kDa	77	48	48	Intestinal endocrine cells
3.67 × 10^−12^	4.26 × 10^−9^	CPNE5	Copine V	489	321	357	B cells, Paneth cells, colonocytes, undifferentiated cells, intestinal endocrine cells
4.00 × 10^−12^	4.26 × 10^−9^	RNF213	Ring finger protein 213	1500	951	863	Colonocytes, T cells, B cells, undifferentiated cells
4.05 × 10^−12^	4.26 × 10^−9^	PREB	Prolactin regulatory element binding	326	255	241	Granulocytes, mucus-secreting cells, T cells, undifferentiated cells
4.61 × 10^−12^	4.56 × 10^−9^	MLKL	Mixed lineage kinase domain-like	456	280	246	Granulocytes, undifferentiated cells
4.78 × 10^−12^	4.59 × 10^−9^	IGFBP5	Insulin-like growth factor binding protein 5	2126	799	527	Undifferentiated cells

List and *P* values generated from a Bayesian t-test of biopsies with endoscopic Mayo subscores > 1 vs. ≤1, as show in Fig. 2.

^a^Cellular expression listed in descending order.

*Used by the Mayo_Prob_1 molecular classifier.

We note that Protein Atlas uses the term ‘enterocytes’ as a general term, and we have used the term ‘colonocytes’ within this table to avoid confusion.

### UC activity transcripts are primarily expressed in inflammatory cells and colonocytes

We analyzed the expression of the top transcripts (increased or decreased in biopsies with high endoscopic Mayo subscore) in colonic tissue using the Human Protein Atlas^32^ (Tables 2 and 3). Many of the top increased transcripts showed elevated expression in granulocytes and colonocytes (e.g. CDC25B, VWF, ZBP1), suggesting nonspecific inflammation and tissue injury. Some top increased transcripts showed significantly increased expression in B or T cells (e.g. PIM2, LOXL2, IFITM2), suggesting adaptive immune activity contributing to the disease.

**Table 3:** Top 30 unique transcripts by *P* value with decreased expression in biopsies with endoscopic Mayo subscore >1 vs. ≤1 (*N *= 128 biopsies).

*P* value	Adjusted *P* value	Gene symbol	Gene name	Expression in biopsies			Cellular expression in colon tissue per human protein atlas^a^
				Mayo > 1	Mayo ≤ 1	Control	
3.29 × 10^−13^	1.38 × 10^−9^	SIAH1	Siah E3 ubiquitin protein ligase 1	367	447	448	Intestinal endocrine cells, colonocytes, B cells
4.74 × 10^−13^	1.43 × 10^−9^	CHPT1	Choline phosphotransferase 1	1580	2349	2549	B cells, undifferentiated cells, colonocytes, T cells, Paneth cells
9.82 × 10^−13^	2.21 × 10^−9^	AKAP1	A kinase (PRKA) anchor protein 1	966	1532	1637	Undifferentiated cells, enterocytes, undifferentiated cells, mucus-secreting cells, Paneth cells
1.27 × 10^−12^	2.61 × 10^−9^	ENTPD5	Ectonucleoside triphosphate diphosphohydrolase 5	113	240	258	Colonocytes, undifferentiated cells, mucus-secreting cells, Paneth cells
1.42 × 10^−12^	2.61 × 10^−9^	TRAK2	Trafficking protein, kinesin binding 2	275	374	387	Colonocytes, undifferentiated cells, intestinal endocrine cells
3.63 × 10^−12^	4.26 × 10^−9^	ARL2-SNX15	ARL2-SNX15 readthrough	277	347	367	NA
3.64 × 10^−12^	4.26 × 10^−9^	PCK1	Phosphoenolpyruvate carboxykinase 1 (soluble)	107	421	618	Colonocytes, undifferentiated cells, mucus-secreting cells, Paneth cells
3.84 × 10^−12^	4.26 × 10^−9^	ETFDH	Electron-transferring-flavoprotein dehydrogenase	293	421	452	Colonocytes, B cells, Intestinal endocrine cells, Paneth cells
4.05 × 10^−12^	4.26 × 10^−9^	CDKN2B-AS1	CDKN2B antisense RNA 1	20	50	81	Colonocytes, Paneth cells, undifferentiated cells
4.48 × 10^−12^	4.52 × 10^−9^	VDR	Vitamin D (1,25- dihydroxyvitamin D3) receptor	648	968	1065	Colonocytes, Paneth cells, undifferentiated cells
5.04 × 10^−12^	4.59 × 10^−9^	PDPK1	3-phosphoinositide dependent protein kinase-1	409	504	508	B cells, T cells, colonocytes, undifferentiated cells, mucus-secreting cells, Paneth cells
5.41 × 10^−12^	4.74 × 10^−9^	ANK3	Ankyrin 3, node of Ranvier (ankyrin G)	190	357	349	Colonocytes, Paneth cells, intestinal endocrine cells
9.55 × 10^−12^	6.27 × 10^−9^	HSD17B11	Hydroxysteroid (17-beta) dehydrogenase 11	733	1154	1147	Colonocytes, Paneth cells, undifferentiated cells, granulocytes
1.06 × 10^−11^	6.70 × 10^−9^	TNNC2	Troponin C type 2 (fast)	24	37	38	Colonocytes, mucus-secreting cells, Paneth cells
1.07 × 10^−11^	6.70 × 10^−9^	ABCG2	ATP-binding cassette, sub-family G (WHITE), member 2	42	90	89	Colonocytes, Paneth cells
1.22 × 10^−11^	7.45 × 10^−9^	SLC26A2	Solute carrier family 26 (sulfate transporter), member 2	278	1350	2035	Colonocytes, undifferentiated cells, Paneth cells
1.30 × 10^−11^	7.70 × 10^−9^	PXMP2	Peroxisomal membrane protein 2, 22 kDa	451	912	978	Colonocytes, undifferentiated cells, Paneth cells, mucus-secreting cells
1.51 × 10^−11^	7.73 × 10^−9^	CDC14A	Cell division cycle 14 A	15	25	27	T cells, colonocytes, Paneth cells, intestinal endocrine cells
1.59 × 10^−11^	7.73 × 10^−9^	ACOX1	Acyl-CoA oxidase 1, palmitoyl	548	1059	1108	Colonocytes, Paneth cells, undifferentiated cells
1.60 × 10^−11^	7.73 × 10^−9^	SNX15	Sorting nexin 15	282	353	375	Granulocytes, undifferentiated cells, intestinal endocrine cells
1.62 × 10^−11^	7.73 × 10^−9^	DHRS11	Dehydrogenase	488	985	1067	Colonocytes, Paneth cells, undifferentiated cells
1.89 × 10^−11^	8.72 × 10^−9^	MGAT4B	Mannosyl (alpha-1,3-)-glycoprotein beta-1,4-N-acetylglucosaminyltransferase, isozyme B	1371	2053	2167	Colonocytes, undifferentiated cells, Paneth cells, mucus-secreting cells
2.09 × 10^−11^	8.89 × 10^−9^	GCOM1	GRINL1A complex locus 1	43	71	71	Paneth cells, colonocytes, undifferentiated cells
2.13 × 10^−11^	8.89 × 10^−9^	VSIG10	V-set and immunoglobulin domain containing 10	178	314	345	Intestinal endocrine cells, colonocytes, undifferentiated cells, Paneth cells, mucus-secreting cells
2.18 × 10^−11^	8.94 × 10^−9^	TRHDE	Thyrotropin-releasing hormone degrading enzyme	16	27	23	Colonocytes, undifferentiated cells, Paneth cells
2.36 × 10^−11^	9.36 × 10^−9^	ACAA1	Acetyl-CoA acyltransferase 1	383	520	559	Colonocytes, Paneth cells, undifferentiated cells
2.56 × 10^−11^	9.61 × 10^−9^	SLC4A4	Solute carrier family 4, sodium bicarbonate cotransporter, member 4	181	428	427	Colonocytes, undifferentiated cells, Paneth cells, mucus-secreting cells
2.66 × 10^−11^	9.84 × 10^−9^	PGRMC1	Progesterone receptor membrane component 1	2864	3565	3897	Colonocytes, undifferentiated cells, Paneth cells, intestinal endocrine cells, mucus-secreting cells
2.88 × 10^−11^	1.03 × 10^−8^	MIR4680	microRNA 4680	374	694	842	NA
3.06 × 10^−11^	1.06 × 10^−8^	RUNDC3B	RUN domain containing 3B	36	76	90	Undifferentiated cells, colonocytes, Paneth cells

List and *P* values generated from a Bayesian t-test of biopsies with endoscopic Mayo subscores > 1 vs. those with ≤ 1, using the samples with the most distinct UC features.

^a^Cellular expression listed in descending order.

We note that Protein Atlas uses the term ‘enterocytes’ as a general term, and we have used the term ‘colonocytes’ within this table to avoid confusion.

Top decreased transcripts showed predominant expression in colonocytes, intestinal endocrine cells, undifferentiated cells, and Paneth cells – indicating a loss of parenchymal function and structural integrity during UC flares (endoscopic Mayo subscore > 1).

### Pathway analysis of UC disease activity emphasize tissue injury and prominence of innate immunity

Top Gene Ontology (GO) terms and Kyoto Encyclopedia of Genes and Genomes (KEGG) pathways (*P* < 0.05) are summarized in Table 4.

**Table 4:** Top 10 pathways from overrepresentation analysis of top 150 genes increased in the comparison of UC biopsies with endoscopic Mayo subscore >1 vs. ≤1 (*N *= 128 UC biopsies).

GO term: biological process	*P* value	GO term: molecular function	*P* value
Extracellular matrix organization	3.5 × 10^−11^	Extracellular matrix structural constituent	5.6 × 10^−11^
Extracellular structure organization	4.4 × 10^−10^	Extracellular matrix structural constituent conferring tensile strength	1.2 × 10^−7^
Connective tissue development	2.8 × 10^−7^	Platelet-derived growth factor binding	2.6 × 10^−6^
Cartilage development	6.3 × 10^−7^	Growth factor binding	5.6 × 10^−5^
Cellular response to amino acid stimulus	3.3 × 10^−6^	Adenosine deaminase activity	1.8 × 10^−4^
Tissue remodeling	7.1 × 10^−6^	Glycolipid binding	1.0 × 10^−3^
Response to amino acid	1.1 × 10^−5^	Protease binding	1.5 × 10^−3^
Collagen metabolic process	1.2 × 10^−5^	Integrin binding	1.8 × 10^−3^
Response to acid chemical	2.1 × 10^−5^	Deaminase activity	3.6 × 10^−3^
Positive regulation of cell adhesion	2.6 × 10^−5^	Hydrolase activity, acting on carbon-nitrogen (but not peptide) bonds, in cyclic amidines	4.3 × 10^−3^

GO term: cellular compartment	*P*value	KEGG pathway	*P*value
Collagen-containing extracellular matrix	1.4 × 10^−10^	Proteoglycans in cancer	3.6 × 10^−4^
Basement membrane	1.5 × 10^−8^	Protein digestion and absorption	4.0 × 10^−4^
Complex of collagen trimers	6.1 × 10^−7^	PPAR signaling pathway	4.8 × 10^−4^
Endoplasmic reticulum lumen	9.3 × 10^−7^	Focal adhesion	1.3 × 10^−3^
Collagen trimer	1.3 × 10^−6^	Choline metabolism in cancer	1.8 × 10^−3^
Extracellular matrix component	6.6 × 10^−6^	PI3K-Akt signaling pathway	2.4 × 10^−3^
Fibrillar collagen trimer	1.2 × 10^−4^	Insulin resistance	3.0 × 10^−3^
Banded collagen fibril	1.2 × 10^−4^	Complement and coagulation cascades	5.2 × 10^−3^
Cytoplasmic vesicle lumen	2.7 × 10^−4^	ECM-receptor interaction	6.0 × 10^−3^
Vesicle lumen	2.8 × 10^−4^	Rheumatoid arthritis	7.6 × 10^−3^

The top GO terms classified as Biological Process (BP) and Cellular Compartment (CC) were mainly associated with matrix and remodeling, e.g. extracellular matrix organization (*P* = 3.5 × 10^−11^) and basement membrane (*P* = 1.5 × 10^−8^). Top Molecular Function (MF) terms were associated with matrix and cellular structure, e.g. extracellular matrix structural constituent (*P* = 5.6 × 10^−11^). Top KEGG pathways included protein digestion and absorption (*P* = 4 × 10^−4^), and peroxisome proliferator-activated receptors (PPAR) signaling pathway (*P* = 4.8 × 10^−4^), which is involved in cellular differentiation, development, metabolism, and tumorigenesis. The TNF signaling pathway was ranked 17th in the KEGG analysis (*P* = 0.003).

**Fig. 3:**
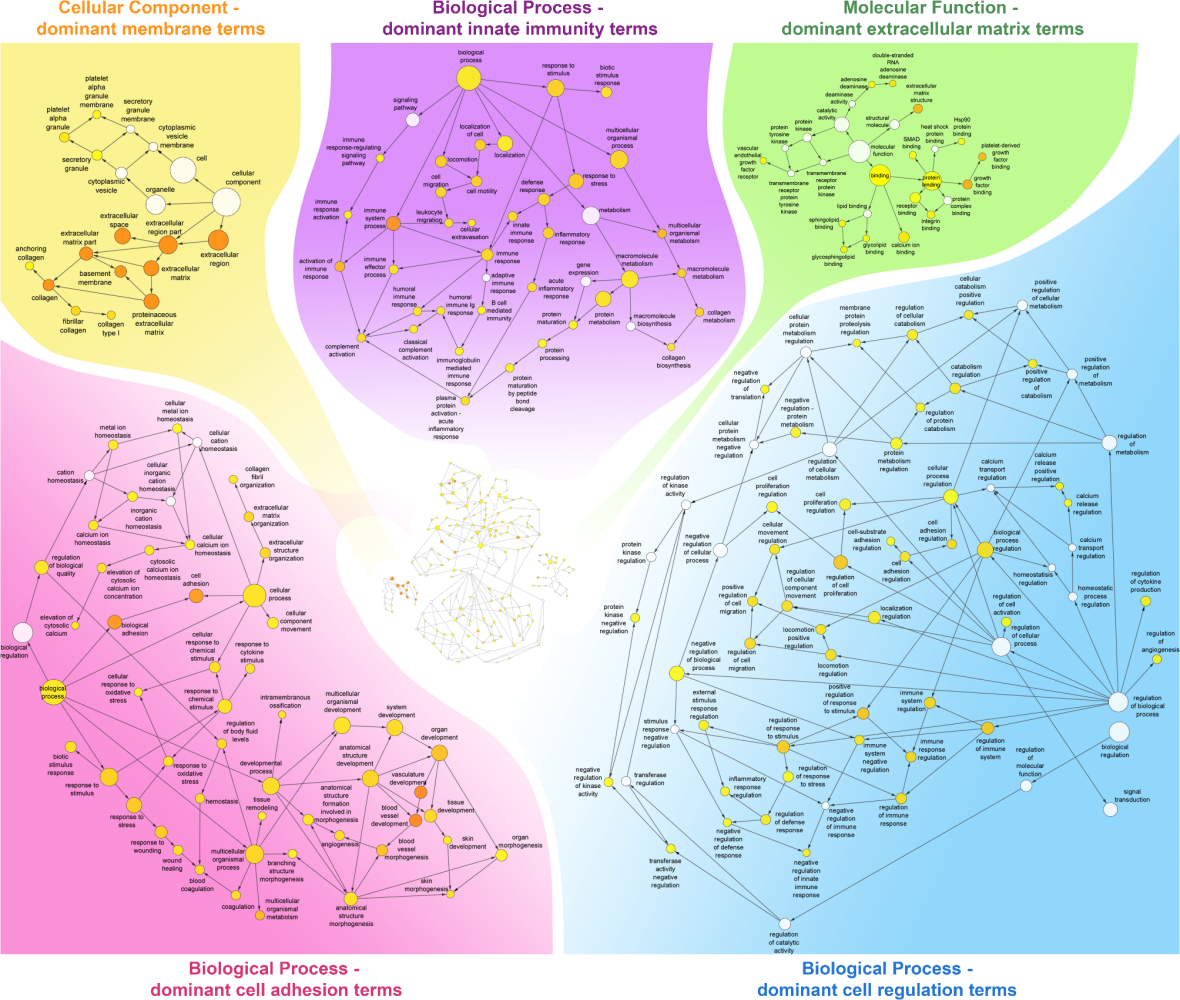
Overrepresentation analysis of top 150 transcripts differentially expressed between biopsies with endoscopic Mayo subscore > 1 vs. ≤ 1. Nodes and edges were generated using Cytoscape and BiNGO. Nodes (circles) are colored by significance (darker colors = higher *P* value), and are grouped according to pathway term type (i.e. biological process – blue, purple, and pink; cellular compartment - yellow, molecular function - green). Redundant relationships were removed to simplify the visual output.

The GO terms are visually represented in Fig. 3. BP terms were separated into three interconnected groups: the purple group, terms mostly associated with inflammation and immunity e.g. ‘innate immune response’; the blue group, terms associated with cell regulation, cytokine production, and protein generation/metabolism e.g. ‘protein metabolism’ and ‘protein maturation’; and the pink group, terms associated with the response to wounding. CC terms (yellow group) were dominated by matrix (e.g. ‘extracellular matrix’), membrane (e.g. ‘basement membrane’), and collagen-associated terms (e.g. ‘fibrillar collagen’). MF terms (green group) focused on matrix structure (e.g. ‘extracellular matrix structure’) and protein binding (e.g. ‘receptor binding’) but also highlighted the significance of heat shock proteins (e.g. ‘HSP protein binding’), implicated in protecting against progression of UC disease^33^.

### Molecular classifiers predict UC disease activity

**Fig. 4:**
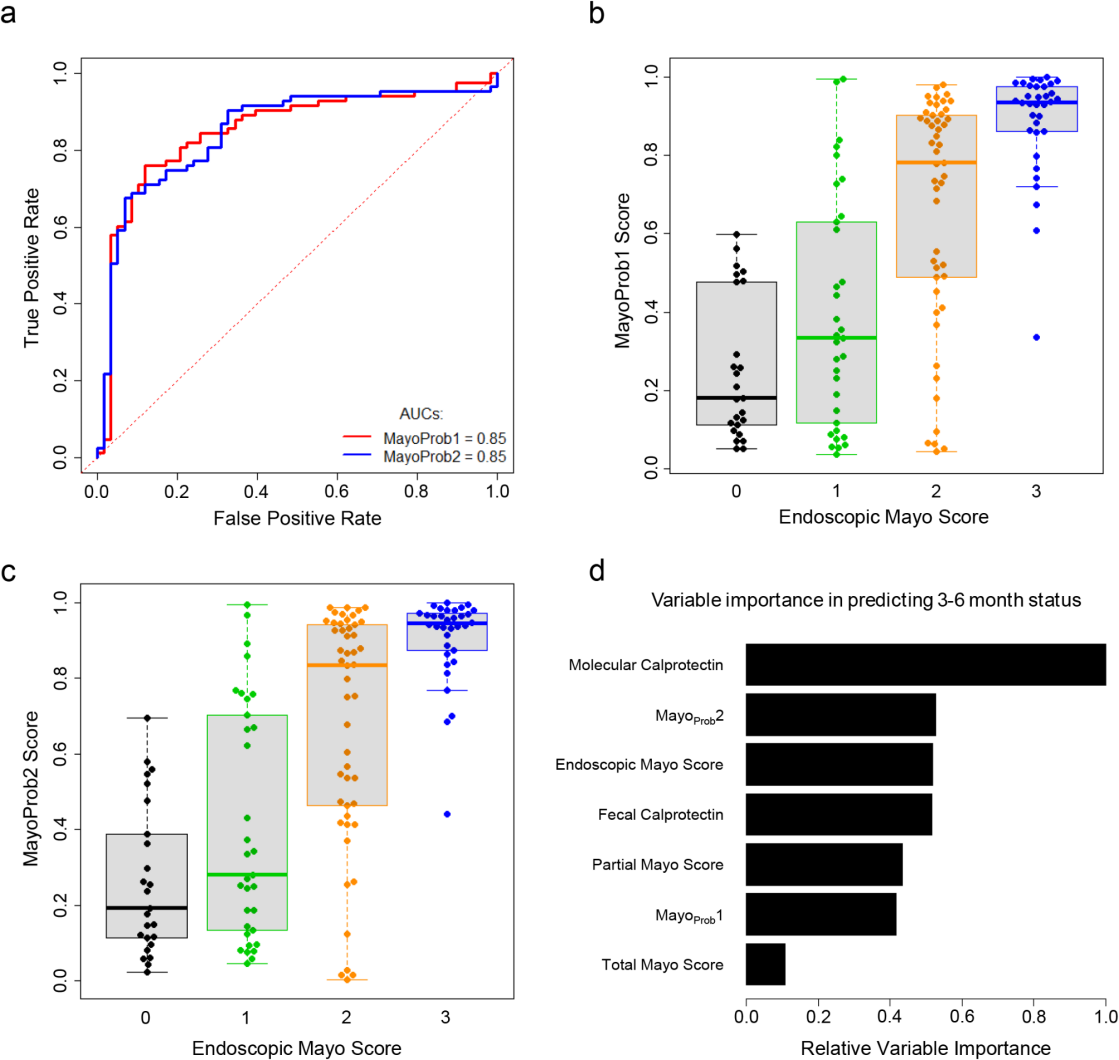
Molecular classifier performance. (**a**) estimated performance of both classifiers shown by area-under-the-curve (AUC). Biopsies were also plotted in beeswarm-boxplots showing their (**b**) Mayo_Prob_1 classifier score vs. endoscopic Mayo subscore, and (**c**) their Mayo_Prob_2 classifier score vs. endoscopic Mayo subscore, showing a mean increase in classifier score as the endoscopic Mayo subscore increases. (**d**) Random forest showing relative variable importance in prediction of 3–6 month post-biopsy status. Molecular (molecular calprotectin, both classifier scores) and standard-of-care variables (fecal calprotectin, partial Mayo score, total Mayo score, endoscopic Mayo subscore) were compared for their importance in predicting 3–6 month post-biopsy patient status.

We developed two molecular classifiers using ten-fold cross-validation and an ensemble of 12 machine learning algorithms for predicting disease activity, trained on the endoscopic Mayo subscore (> 1 vs. ≤1). The first classifier, Mayo_Prob_1, used only UC biopsies (*N* = 128), and the second, Mayo_Prob_2, used a slightly larger population of both UC and IBDU biopsies (*N* = 141). Both classifiers predicted endoscopic Mayo subscore > 1 with an area-under-the-curve (AUC) of 0.85 (Fig. 4A).

Classifier scores for both Mayo_Prob_1 (Fig. 4B) and Mayo_Prob_2 (Fig. 4C) increased with a rise in the endoscopic Mayo subscore in the biopsies.

### Molecular scores correlated with clinical variables

We assessed the correlation between the two molecular classifiers (Mayo_Prob_1 and Mayo_Prob_2), calprotectin transcript set score (MCalpro, the geometric mean of the standardized expression scores of the S100A8 and S100A9 transcripts, see Methods), and several clinical variables: total Mayo score, endoscopic Mayo subscore, and fecal calprotectin (Table 5). Clinical variables were ordinal and categorical while molecular scores were continuous numbers.

**Table 5:** Spearman correlation coefficients – molecular classifier scores compared with clinical features in UC and IBDU biopsies (*N* = 141 biopsies).

	Clinical features^a^						
	Total Mayo score at biopsy (0–12)	Partial Mayo score at biopsy (0–9)	Physician’s global assessment at biopsy (0–3)	Endoscopic Mayo subscore (0–3)	Fecal calprotectin at biopsy		
Mayo_Prob_1 classifier score (continuous variable)	**0.61** **(*P*** **= 8.1 × 10**^**−14**^**)**	0.36 (*P* = 2.2 × 10^−5^)	**0.67** **(*P*** **= 2.2 × 10**^**−12**^**)**	**0.67** **(*P*** **< 2.2 × 10**^**−16**^**)**	0.47 (*P* = 0.004)		
Mayo_Prob_2 classifier score (continuous variable)	**0.61** **(*P*** **= 1.7 × 10**^**−13**^**)**	0.37 (*P* = 1.0 × 10^−5^)	**0.65** **(*P*** **= 9.0 × 10**^**−12**^**)**	**0.67** **(*P*** **< 2.2 × 10**^**−16**^**)**	0.40 (*P* = 0.01)		
Molecular Calprotectin transcript set score	**0.62** **(*P*** **= 6.5 × 10**^**−14**^**)**	0.22 (*P* = 0.009)	**0.67** **(*P*** **= 1.25 × 10**^**−12**^**)**	**0.59** **(*P*** **= 1.3 × 10**^**−14**^**)**	**0.63** **(*P*** **= 6.4 × 10**^**−5**^**)**		

^a^Correlations > 0.5 are bolded.

The Mayo_Prob_1 classifier score correlated with endoscopic Mayo subscore (correlation coefficient 0.67), the physician’s global assessment at biopsy (0.69), and the total Mayo Score (0.61). The Mayo_Prob_2 classifier correlations were similar to those of Mayo_Prob_1.

MCalpro correlated with fecal calprotectin (correlation coefficient 0.63), endoscopic Mayo subscore (0.59), total Mayo score (0.62), and physician’s global assessment (0.67).

### Molecular features correlated only moderately overall with the partial Mayo subscore

Molecular features correlated with future patient status. Of the 141 biopsies (colonoscopies), 80 patients had follow-up at 3–6 months (Supplementary Table S1). To assess the relationship between molecular UC disease features at time of biopsy to short-term clinical outcomes, we developed a status code classification that summarized the change between the patient’s disease on the day of biopsy and 3–6 months post-biopsy: patients with poor outcomes (status code > 1) vs. those with favorable outcomes (status code ≤ 1) using t-tests (Table 6). This revealed significant differences in MCalpro, Mayo_Prob_1, and Mayo_Prob_2 scores (*P* = 0.01, 0.005, and 0.002, respectively). The endoscopic Mayo subscore also differed significantly, though with a weaker *P* value (*P* = 0.02) than the molecular scores. Other clinical variables did not demonstrate differences between those with poor or favorable outcomes.

We compared the importance of standard-of-care clinical variables (endoscopic Mayo subscore, partial Mayo score, total Mayo score, and fecal calprotectin) to the MCalpro and classifier scores in random forests for the prediction of the 3–6 month status code (Fig. 4D). The most important variables for this prediction were molecular features (MCalpro and the Mayo_Prob_2 classifier score).

**Table 6:** Relationship between various scores at time zero and future status code (assessed at 3–6 months post-biopsy, status code >1 vs. ≤1) for all UC and IBDU biopsies (*N *= 141 biopsies).

	Variable	Mean score at time zero		
		Biopsies with good future status: status code ≤ 1	Biopsies with poor future status: status code > 1	T-test *P* value
Clinical features	Total Mayo score	6.11	6.81	0.35
	Physician’s global assessment	1.65	1.94	0.29
	Partial Mayo score	3.26	3.75	0.51
	Endoscopic Mayo subscore	1.89	*2.33*	**0.02**
	Fecal calprotectin (ug/g)	758	1247	0.16
Molecular features	Mayo_Prob_1 classifier score	0.63	*0.80*	**0.005**
	Mayo_Prob_2 classifier score	0.64	*0.82*	**0.002**
	Molecular Calprotectin transcript set score	2.49	*3.71*	**0.01**

Bold indicates significance at *P* < 0.05.

Italic indicates the biopsy group with the highest mean score of the row when *P* is significant.

*P* value was assessed using Welch’s t-test.

### Molecular scores improve the prediction of future patient status in logistic regression models

We used logistic regression to compare two models predicting 3–6 month post-biopsy clinical status (Table 7). Model 1 included standard-of-care variables (fecal calprotectin, partial Mayo score, and endoscopic Mayo subscore). Model 2 included the same standard-of-care variables plus molecular variables: MCalpro, Mayo_Prob_1, and Mayo_Prob_2. The full model with both molecular and clinical features was better than the model using clinical features alone (*P* = 0.035).

**Table 7:** Predicting 3–6 month post-biopsy status considering only standard-of-care clinical features and molecular features developed in these analyses (UC and IBDU, *N *= 141 biopsies).

Predictor variable(s)		*P* value	AUC
Total Mayo score		0.36	0.59
Endoscopic Mayo subscore		**0.03**	0.67
Fecal calprotectin		0.16	0.72
Molecular calprotectin		**0.01**	0.68
Mayo_Prob_1 classifier		**0.007**	0.63
Mayo_Prob_2 classifier		**0.01**	0.63

Model comparison		*P *value^a^	Interpretation
Endoscopic Mayo subscore + total Mayo score + Fecal calprotectin	Endoscopic Mayo subscore + total Mayo score + fecal calprotectin + Mayo_Prob_1 + Mayo_Prob_2 + MCalpro	**0.035**	Molecular features add to clinical features
Mayo_Prob_1 + Mayo_Prob_2 + MCalpro	Endoscopic Mayo subscore + total Mayo score + fecal calprotectin + Mayo_Prob_1 + Mayo_Prob_2 + MCalpro	**0.037**	Clinical features add to molecular features

^a^Likelihood ratio test; AUC, area-under-the-curve.

Significant values are in bold.

## Discussion

This study was designed to define the molecular processes occurring in the mucosa of active UC and assessed whether molecular classifiers could add to the clinician’s ability to predict disease trajectory. We found that the molecular landscape of UC is dominated by innate immune processes, particularly complement factors, and transcripts associated with parenchymal tissue injury. Transcripts were highly expressed in colonocytes and granulocytes, but less so in T and B cells. Molecular classifiers were developed using transcripts most differentially expressed between biopsies with high and low disease activity (endoscopic Mayo subscore > 1 vs. ≤1). A molecular calprotectin score (using S100A8 and S100A9 transcripts) showed strong relationships to fecal calprotectin proteins at time of biopsy. Notably, the molecular classifiers (Mayo_Prob_1 and Mayo_Prob_2) and the molecular calprotectin score was important for the prediction of poor patient status at 3–6 months post-biopsy, and both added significantly to a model predicting 3–6-month disease status vs. conventional UC disease variables (as per logistic regression). This indicates molecular classifiers have the potential to add to clinical assessment at time of biopsy by anticipating future disease status during standard-of-care management. This may help identify patients who will require intensive therapy early on (i.e. escalated dosing, adjunct steroids, or change of therapy).

Because patient response to UC therapies such as anti-TNF, anti-IL12/23, anti-integrin alpha 4 beta 7, and JAK inhibition remains highly variable, prediction at time of biopsy of patients who may have a poor outcome is important – not only to indicate the need for intensified therapeutic approach, but also for escalating monitoring approaches. Endoscopic and histologic healing have presented alternative endpoints for predicting ‘good’ vs. ‘poor’ outcomes in patients, but both represent *outcomes* that eventually become predictive. Conversely, molecular scoring is predictive *at the time of initial endoscopy*: endoscopic and histologic healing are the targets. As initial assessment tools, both histology and endoscopy remain challenging and have not been standardized: endoscopic appearance has a less than optimal relationship with outcomes^34^, while histologic scoring requires complete histology performed at every endoscopy (not currently the standard-of-care at every center), is subject to interobserver variation^35^, and is heterogenous across centers^36^.

Machine-learning-derived classifiers present the advantage of being rapidly accessible and highly reproducible when the molecular platform is standardized: analyzing the same mRNA sample twice on microarrays gives the same results with > 99% reproducibility^21^. Classifier output (scores) are continuous numerical values, capable of describing greater heterogeneity within the diverse UC patient population compared to any ordinal endoscopic or histologic assessment. A molecular report with all scores for a new biopsy can be generated within 48 h of biopsy^37^, faster than average histologic or fecal calprotectin testing. Overall, classifiers and molecular calprotectin scores add a new, highly reproducible dimension to conventional variables, potentially replacing fecal calprotectin when endoscopy is performed, leaving fecal calprotectin for non-endoscopic assessment.

The molecular landscape of UC indicates multiple innate immune processes but is not definitive for a primary role for adaptive immunity e.g. cognate effector T cell activity, despite considerable interest in T cell processes in UC^38–40^. While our previous work found that lymphoplasmacytic infiltrate in UC biopsies correlated with the molecular disturbance^14^ and is a predictor of relapse in patients^41^, this may be related to the response-to-wounding and chronicity, rather than active disease. Mucosal injury leads to a breakdown in the epithelial barrier, and microbiota and bacterial products likely perpetuate an ongoing neutrophilic inflammatory process, even if the initial injury process has diminished. One possibility is that initiating and sustaining mechanisms are separate: injury to the epithelium – e.g. a cognate T cell event – can be remembered through reprogramming of epithelial stem cells (EpSCs, through chromatin changes in genomic regions associated with inflammation), rendering the mucosa susceptible to otherwise innocuous inflammatory stimuli^30,31^. Of interest, inflammasome transcripts correlating with UC activity are among those encoded by genes in open chromatin domains induced by damage in EpSCs (e.g. AIM2, IL-1B, and CASP1)^30,31^. This finding is compatible with the association between UC activity and the inflammasome, including strong associations of NOD2, IL18, and caspases with UC activity^42^. Thus cognate T cell-mediated autoimmunity could set the stage for continuing UC activity^39,43–45^ via alterations in EpSCs, reprogramming the epithelium to be vulnerable to local influences such as microbes and their products that are usually innocuous^30,31,46^. This model may explain the inconsistent efficacy of empirically derived therapies for UC, and why such therapies differ strikingly in their efficacy from a pure cognate T cell driven disease process, like organ transplant rejection.

Limitations to this study include the use of standard-of-care management rather than a strict management protocol, but this also provides a real-world environment for our conclusions in preparation for larger studies exploring specific therapeutic protocols or various disease phenotypes, creating a future in which the choice of therapy is guided by the molecular features, as is often the case in oncology. Machine learning creates precise algorithms even when trained on gold-standard clinical assessments that are inherent with errors or variability^20^. While we selected microarrays for the many benefits of this technology, we acknowledge the inherent limitations compared to other possible methods (e.g. RNA sequencing) for analysis: microarrays limited our analyses to the 19,462 unique transcripts present on the chip, in some cases may limit sensitivity for low-expression genes, and does not provide the same level of data as RNA sequencing for machine learning applications. Therapeutics were not used as a variable in these analyses due to heterogeneity preventing meaningful analysis but may be considered in the future with a larger dataset and more defined groups of therapeutic strategies. Future analyses looking at later time points will better define the predictive ability of molecular scores, and we also plan to assess cases with discrepancy between molecular scores, endoscopy activity, and fecal calprotectin, with a further goal of developing a prospectively followed cohort, with set time points of clinical, biochemical, and endoscopic assessment. Multivariable analysis was unavailable due to the limited dataset size.

We acknowledge a desire for less invasive tests (e.g. of peripheral blood), but this must be balanced with deficiencies in this approach. Such tests may offer an improved safety profile with more frequent monitoring, but lose specificity as they correlate with different disease processes^19,47–49^. Recent development of a blood-based gene-expression test for IBD showed some improvement over C-reactive protein, but did not add additional information to fecal calprotectin or endoscopy^50^. Our goal was to build on and enhance the accepted gold standard-of-care – endoscopic assessment and histology.

Management of UC is based on assessments that have suboptimal granularity and reproducibility – standardized molecular assessments can potentially address this need. Most standard-of-care methods are either subjective with high interobserver variation (Mayo scores, physician global assessment), or serve as a binary tool for predicting disease severity (fecal calprotectin) rather than as a means for predicting patient outcomes. The molecular phenotype of UC assessed using standardized microarrays and machine learning classifiers offers a granular approach to assessing disease severity and activity. Furthermore, the molecular toolset developed here provides an additional source of rapidly available, reproducible data when making management decisions, added to standard-of-care assessment, and has the potential for improving patient outcomes.

## Methods

### Patients, biopsy collection, and diagnoses

Patients (*N* = 120) age ≥ 18 years were prospectively enrolled based on an established clinical diagnosis of UC during standard-of-care colonoscopies. 1–4 bites per patient were collected from the most endoscopically inflamed area (as determined by the endoscopist), combined as a single ‘biopsy’ for a total of 141 biopsies, as per protocols at the Center of Excellence for Gastrointestinal Inflammation and Immunity Research (CEGIIR, University of Alberta Hospital, Edmonton, Canada) and at Cedars-Sinai Medical Center (Los Angeles, USA). During patient enrollment, 13 (of the 120) patients underwent more than one colonoscopy at separate time points, hence why a total of 141 biopsies were included. Normal biopsies were also collected from 17 non-UC patients undergoing colonoscopies with no known history of IBD or other gastrointestinal disease. These control biopsies were only used in select analyses, when indicated. All biopsies were placed in RNA*later*™, and stored at −20 °C for isolation of RNA.

Demographics (age, sex, date of diagnosis), disease status (partial Mayo score)^51–53^, medications at the time of biopsy, and endoscopic data (extent of disease, endoscopic Mayo subscore for colon segments) were all collected at the time of endoscopy and verified with the electronic medical records for all patients. All IBD medications were permitted within this population, including 5-aminosalicylic acid formulations, corticosteroids, immunomodulators, antibiotics, and advanced therapies (biologics and small molecules).

All diagnoses were established by experienced IBD gastroenterologists using standard-of-care methods (Supplementary Table S2)^14^.

### Study approval

The study was reviewed and approved by the University of Alberta Health Research Ethics Board (HREB) Biomedical Panel (Edmonton, Canada), and Cedars-Sinai Institutional Review Board (Los Angeles, USA). Participants provided written informed consent to research study staff and received a copy of their signed informed consent. This study was performed in accordance with relevant guidelines, regulations, and with the Declaration of Helsinki.

### Biopsy processing

Biopsies in RNA*later*™ were transferred to the Alberta Transplant Applied Genomics Center (ATAGC, Edmonton, Canada) for processing. Total RNA was extracted, cleaned, and labeled using established methods^17^. Extracted RNA was high quality and yield (mean RNA integrity number (RIN) 7.7, average yield 1.78 µg). Purified total RNA was labeled with the IVT Express labeling kit or IVT Plus labeling kit (Affymetrix, Santa Clara, USA) and hybridized to human PrimeView arrays (Affymetrix) according to manufacturer protocols^21^.

The Affymetrix PrimeView GeneChip microarrays include 19,462 unique (nonredundant) transcripts from 49,495 probe sets available on the array. Microarrays were scanned, ‘CEL’ files were obtained using GeneChip Operating Software (Affymetrix), and robust multiarray averaging was used to normalize the CEL files^17^. Once all CEL files were available, a correction factor was calculated to normalize CEL file expression data between biopsies processed using different labeling kits.

### Overrepresentation analysis

Overrepresentation of UC activity-associated transcripts in GO and in KEGG pathways was analyzed using the “enrichGO” function from the “clusterProfiler”^54^ package in R^55^.

Pathway terms were visualized using Cytoscape^56^ and the associated BiNGO^57^ and stringAPP^58^ applications. The top 150 transcripts increased in high endoscopic Mayo subscore biopsies were used (all had an adjusted *P* < 0.05). This allowed many significant transcripts to be represented, but simplified the visual result by limiting the number of transcripts used.

### Human protein atlas

Cellular expression in colon was assessed using the Human Protein Atlas^32^. Each transcript was searched, and the single-cell expression examined in colonic tissue. The top five cell types were listed when applicable, otherwise only reported cellular expression was listed.

### Classifier development

A classifier was developed for predicting disease activity (endoscopic Mayo subscore > 1 vs. ≤1). Upon chart review, it was revealed that a minority of patients were originally labeled with indeterminate features (clinically and endoscopically) indicating a diagnosis of inflammatory bowel disease unclassified (IBDU), so the first classifier was trained on a population that excluded these samples (Mayo_Prob_1). The second classifier (Mayo_Prob_2) used the larger dataset of all biopsies (UC and IBDU), as patients had complete endoscopic Mayo subscores and had been clinically managed as UC. Both classifiers were used throughout these analyses to best represent real-life clinical practice in UC and to observe the benefit of increased statistical power in classifier development.

Using 10-fold cross validation we randomly split the biopsy set used for each classifier (Mayo_Prob_1, Mayo_Prob_2) into a training set (90% of all samples) and a test set (10% of all samples). This was repeated 10 times for all 10 folds per previously established protocols, resulting in 10 different t-tests and 10 different lists of top 20 probe sets^59^. This process is summarized in Supplementary Fig. S1. The classifier was trained on binary classes of endoscopic Mayo subscores (disease class = scores > 1, and without disease = scores ≤ 1) as reported by the endoscopist during endoscopy and generated ‘probability of active disease’ estimates ranging from 0.0 to 1.0.

Each algorithm used the top 20 probe sets selected by t-test between the classes. For stability, each classifier was trained in 12 machine learning algorithms, and the median of all 12 algorithms was used as an ensemble classifier score, as previously published^20^.

The 12 different machine learning algorithms were: linear discriminant analysis (lda), regularized discriminant analysis (rda), mixture discriminant analysis (mda), flexible discriminant analysis (fda), gradient boosting machine (gbm), radial support vector machine (SVMR), linear support vector machine (SVML), random forest (rf), C5.0, neural networks (nnet), Bayes glm (bayesglm), and generalized linear model elastic-net (glmnet). The output value of the classifier was the median score from 12 separate machine learning algorithms, as this provides increased stability of the estimate.

### Development of a transcript set score for calprotectin expression in the biopsy

Transcript set scores are a set of transcripts represented on the microarray and are chosen by the end user to represent a particular biology or disease mechanism. A transcript set score is calculated as the geometric mean of the standardized expression scores (standardized against a control population). We developed a transcript set to represent calprotectin-associated expression, using the probe sets for S100A8 and S100A9 (as the gene products of S100A8 and S100A9 represent the calprotectin heterodimer). This became the molecular calprotectin score (MCalpro) – the geometric mean expression of S100A8 and S100A9 in a defined population vs. the 17 normal (control) colon biopsies.

### Development of a status code to describe disease outcomes 3–6 months post-biopsy

To assess the relationship between molecular UC disease features at time of biopsy to short-term clinical outcomes, we developed a status code classification that summarized the change between the patient’s disease on the day of biopsy and 3–6 months post-biopsy (Supplementary Table S3). The status code was assigned by an IBD clinician (BPH), who was blinded to the molecular data. ‘Good’ outcomes were assigned if the patient was in remission 3–6 months post-biopsy (status 0) or had significant improvement with some ongoing disease activity (status 1). ‘Poor’ outcomes were assigned if the patient was having ongoing disease activity with no response to clinical management (status 2) or worsening disease activity (status 3).

Derivation of the status code (outcome) was based on disease activity and severity (including biochemical parameters (C-reactive protein, fecal calprotectin), endoscopic Mayo subscores, partial Mayo scores), any change in therapy shortly after the biopsy, therapy response from the time of the biopsy to the 3–6-month reassessment, and assessment of current disease as documented by the endoscopist or treating physician. In cases where follow-up endoscopic evaluation did not occur within 3–6 months, the partial Mayo score was used. The electronic medical record was reviewed to ensure the patient did initiate the prescribed new or escalated treatment(s) between the time of biopsy and the 3–6 month reassess. Total and partial Mayo criteria are described in Supplementary Table S4.

This scoring system was created to represent common clinical assessments for a treating physician during standard-of-care reassessment of patients. A 3–6 month time frame was used to provide a reasonable window for disease improvement and response to any management changes shortly after biopsy.

### Authors

All authors had access to the study data and had reviewed and approved the final manuscript.

###  Statistics

Statistical analysis and graphics were done in the “R” software package, version 4.0^55^ with various libraries from Bioconductor 3.2^60^, and in Microsoft Excel version 16 (Redmond, WA). Differentially expressed genes were generated using the ‘limma’ Bioconductor package^61^. Significance of probe set expression is given as unadjusted *P* values (Bayesian t-test), except in cases where the false discovery rate (FDR) is specified. Volcano plots were generated using Excel and colored by previously assigned transcript sets of interest.

## Electronic supplementary material

Below is the link to the electronic supplementary material.

Supplementary Material 1

## Data Availability

Microarray (CEL) files relevant to this study will be made available on the Gene Expression Omnibus (GEO) website when the paper is published. Deidentified data and methods can be shared on reasonable request to the corresponding author.
