# Supplementary material for: Relating the molecular phenotype of ulcerative colitis to the clinical course
Source: Sci Rep. 2025 Mar 11;15:8342. doi: 10.1038/s41598-025-90618-8 (PMC11894109; doi:10.1038/s41598-025-90618-8)
Supplement: Supplementary file 1 — Supplementary Material 1 [file 41598_2025_90618_MOESM1_ESM.pdf]

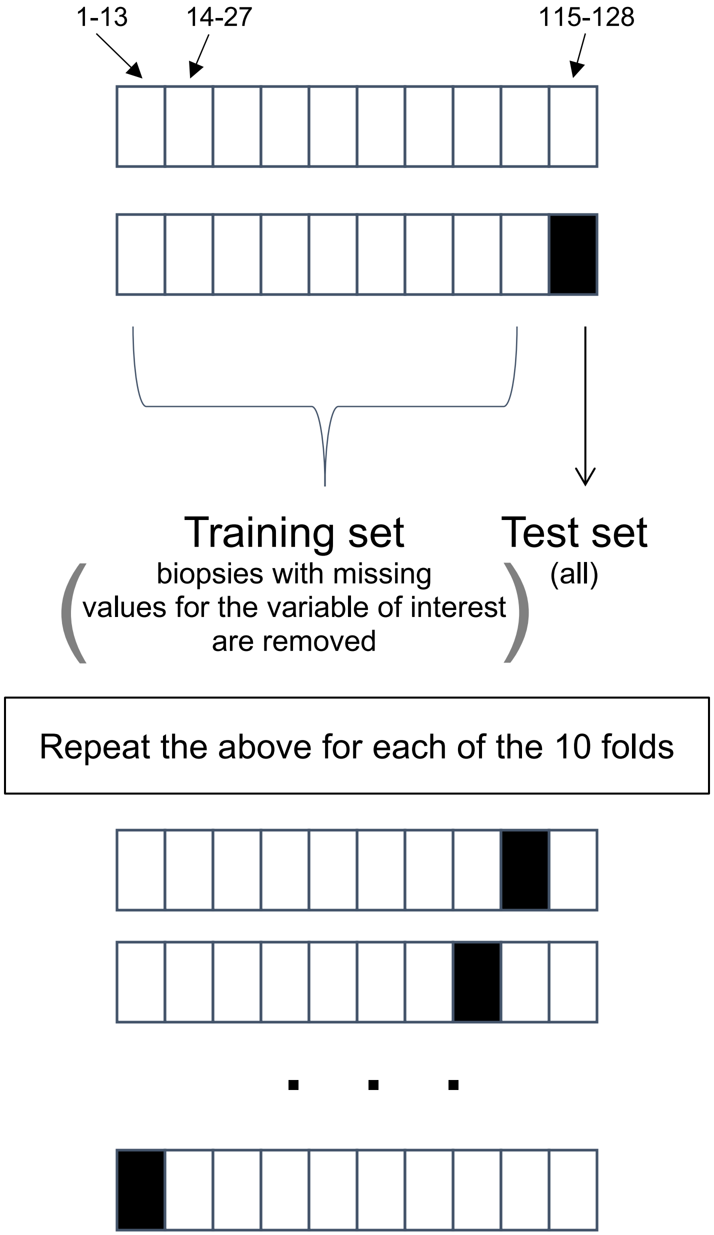

Supplementary Figure S1. Diagram showing the methods used for classifier development. Nine-tenths of the samples are randomly assigned to a training set. The training set is used to build a predictive equation using one of the selected algorithms e.g. linear discriminant analysis (lda). The equation uses the top 20 probe sets (by *P* value) for distinguishing high from low values for the diagnosis/feature, using Welch's t-test. All test set samples are then assigned a score by multiplying the 20 weighted coefficients derived from the training set by the test set expression values of the 20 probe sets. This process is repeated for each of the 12 selected algorithms, and the median of all 12 predicted scores is taken as the final classifier score for that sample. Each of the samples ends up with a single predicted score, from the one time it was in a test set.

Supplementary Table S1. Type of follow-up at 3-6 months post-biopsy collection.

Type of follow-up	N (total = 141)
Colonoscopy follow-up	28
Clinic follow-up	52
Follow-up >6 months post-biopsy*	41
Cedars-Sinai patients (3-6 month) follow-up data not available)	20

*Many patients had delayed follow-up (>6 months) due to COVID-19.

Supplementary Table S2. Diagnostic criteria for UC and IBDU used in this study (N=141 biopsies)

Abbreviation	# biopsies in population	Diagnosis	Definition	Criteria
**UC**	**128**	**Ulcerative colitis**	- This has been defined as patients diagnosed with UC regardless of disease activity (Endoscopic Mayo subscore 0-3 included).<br>- Diagnosed by an expert at a credible university.<br>- Main samples considered in these analyses.	- Chronic inflammation of the colonic mucosa with involvement of the rectum and spread more proximally in the setting of symptoms of rectal bleeding, urgency and diarrhea in the absence of infection.
**IBDU**	**13**	**Inflammatory Bowel Disease Unclassified**	- Managed clinically as UC.<br>- All symptoms and endoscopy scored as UC.	- Chronic inflammation of the colonic mucosa that had features of UC with some atypical features i.e. non-confluent inflammation (patchy disease), lack or minimal rectal bleeding.<br>- No definitive features of Crohn’s disease (granuloma, fistula, small bowel inflammation).

Supplementary Table S3. Status code describing change in patient course between time of biopsy and 3-6 months post-biopsy

Status Code value	Description	Binary version of status code used in this analysis:
0	remission	0 (good outcome)
1	improvement w/ ongoing activity	
2	ongoing activity, with no response	1 (bad outcome)
3	worsening activity/colectomy	

Supplementary Table S4. Description of total Mayo score and subscores as used in clinical practice for all biopsies throughout this study.

**Total Mayo score as used in these analyses**		
**Score Parameter**	**Subscores as evaluated by a clinician**	**Corresponding Scores**
Stool Frequency[a] (/day)	Normal number of stools	0
	1-2 more than normal	1
	3-4 more than normal	2
	≥5 more than normal	3
Rectal bleeding[a] (most severe of the day)	None	0
	Streaks of blood in stool in less than half the cases	1
	Obvious blood with stools in most cases	2
	Blood alone passes	3
Endoscopic findings[b]	Normal mucosa or inactive disease	0
	Mild activity (erythema, decreased vascular pattern, mild friability)	1
	Moderate activity (marked erythema, lack of vascular pattern, friability, erosions)	2
	Severe activity (spontaneous bleeding, large ulcerations)	3
Physician's global assessment*	Normal	0
	Mild disease	1
	Moderate disease	2
	Severe disease	3

**Interpretation of the sum total Mayo score**	
**Sum total of all scores**	**Interpretation**
0-2	Remission (provided that no single subscore is >1)
3-5	Mild disease activity
6-10	Moderate disease activity
>10	Severe disease activity

Score assignments and coding adapted from:

1. Schroeder KW, Tremaine WJ, Ilstrup DM: Coated oral 5-aminosalcylic acid therapy for mildly to moderately active ulcerative colitis. N Eng J Med 1987; 317 (26): 1625-1629.
2. Rutgeerts P, Sandborn WJ, Feagan BG, Reinisch W, et al. Infliximab for induction and maintenance therapy for ulcerative colitis. N Engl J Med. 2005; 353 (23): 2462-2476.
3. Dhanda AD, Creed TJ, Greenwood R et al. Can Endoscopy Be Avoided in the Assessment of Ulcerative Colitis in Clinical Trials? Inflamm Bowel Dis 2012; 18 (11): 2056–2062.

[a]These features make up the partial Mayo subscore.

[b] This feature is the endoscopic Mayo subscore.
